# Supplementary material for: Protein Sumoylation Is Crucial for Phagocytosis in Entamoeba histolytica Trophozoites
Source: Int J Mol Sci. 2021 May 27;22(11):5709. doi: 10.3390/ijms22115709 (PMC8198320; doi:10.3390/ijms22115709)
Supplement: Supplementary file 1 [file ijms-22-05709-s001.zip › ijms-1166651-supplementary.pdf]

International Journal of *Molecular Sciences*

MDPI

Supplementary Material

Table S1. Putative SUMOylation sites in published proteins involved in *Entamoeba histolytica* phagocytosis.

Function	Protein	Number access	Position	Peptide/sequence	Score	Site	Reference
Retromer	Vacuolar sorting protein 29	EHI_025270	3 - 7	*****ML VLVIG DFHVPHR	64.072	SUMO Interaction	[1]
			56 - 60	LRTLARE VHVVK GDFDEMQ	65.27	SUMO Interaction	
			60	AREVHVVKGDFDEMQ	44.952	Sumoylation	
			110 - 114	QRQLDVD ILITG HTHKLET	60.6	SUMO Interaction	
			173	TLVDGSVKCERVDFN	45.097	Sumoylation	
	Vacuolar sorting protein 26	EHI_062490	9-13	AFLFGTP IQIDI LLDNDHE	72.761	SUMO interaction	[1]
			40	TEIPIYMKNEDVNGK	30.527	Sumoylation	
			145 - 149	GLSKEQE IWVIN YQDEPTK	74.151	SUMO interaction	
			238	LMDGAPVKGESMPIR	43.592	Sumoylation	
			342	PQQSEEPKEEIKEPV	21.38	Sumoylation	
			362	QSIQEEPKEEIKEEK	21.38	Sumoylation	
			366	EEPKEEIKEEKKEEP	45.815	Sumoylation	
			374	EEKKEEPKEEVKEEI	21.902	Sumoylation	
			378	EEPKEEVKEEIKEEP	42.644	Sumoylation	

			382	EEVKEEIKEEPKEEV	47.766	Sumoylation	
			386	EEIKEEPKEEVKEKP	21.902	Sumoylation	
			390	EEPKEEVKEKPKAID	39.231	Sumoylation	
			406	SSFIQDDKQDDDNLF	37.443	Sumoylation	
	Vacuolar protein sorting 35	EHI_002990	86	TPLFMFLKDEYEGSL	25.295	Sumoylation	[1]
			309	KVLVNFIKSEETTLD	49.647	Sumoylation	
			462 - 466	SNMLEVI IDLIS KVQDQPD	59.445	SUMO interaction	
			476	QDQPDDIKEEEIGVD	48.626	Sumoylation	
			556	AISKMVAKLEHYVLA	17.505	Sumoylation	
ESCRT	Tumor susceptibility gene 101 protein putative	EHI_178530	35	TSVMQYYKFSATVRT	25.383	Sumoylation	[2]
			47 - 51	VRTYADG IILAS LVGTIPI	56.467	SUMO interaction	
			67 - 71	YRGSQFC LPLCI MYPYDYP	56.376	SUMO interaction	
			283	TLTPLNTK*******	38.49	Sumoylation	
	Vacuolar sorting protein VPS4	EHI_118900	78	MISGERVKSDDPDKE	45.994	Sumoylation	[2]
			128 - 132	EALQEAV ILPIK FPQLFTD	59.356	SUMO interaction	
			231	TEASRRVKTEFLVQM	41.814	Sumoylation	
Vesicular trafficking	EhRabB	EHI_181240	82 - 86	YYRGSQG ILIVY DVSNFDS	63.59	SUMO Interaction	[3]
			105	TYWIGELKKENVDGI	28.197	Sumoylation	
	EhRab7	EHI_001870	83-87	FRGTDGC VLVCS VTSIESF	60.125	SUMO interaction	[4]
			120	PFVAVANKSDLDPIE	39.109	Sumoylation	
Actin related proteins	Actin related protein 2/3 complex, subunit 4	EHI_030820	51 - 55	KEVIFKP VVIVR VPSEPSG	61.242	SUMO interaction	[5]

	EhABPH (actin-binding protein)	EHI_122800	422	RRSSVSLKPEHMKSI	26.543	Sumoylation	[6]
			523 - 527	TEEIEGK VGIVI EIDEESE	63.088	SUMO interaction	
			586	KQETKEVKEEEKTQR	44.223	Sumoylation	
			674 - 678	QLTSKGN VIHLA CKNEEEL	62.786	SUMO interaction	
			698	ACTPNNLKGEEADEE	24.332	Sumoylation	
			753 - 757	NRTDSTP ILKIY LKDIENI	61.532	SUMO interaction	
			787	KGNKHEFKTEECKAW	18.512	Sumoylation	
			795	TEECKAWKEEIMERK	24.947	Sumoylation	
			930	VILLTHGKDDRETDM	37.458	Sumoylation	
			973 - 977	QDKEEPE IIRIY RIVHNEK	61.19	SUMO interaction	
			1236 - 1240	KIFKGKF IVHLG GYSEYYD	63.428	SUMO interaction	
			1297 - 1301	VFKTIKG IEVIE GKDSNEE	67.442	SUMO interaction	
			1426	ERGLKEVKIEQENEE	42.552	Sumoylation	
			1467 - 1471	PVCQKKE IELIT QVIDGTF	60.742	SUMO interaction	
			1486	NEIDPALKNEISVEG	25.939	Sumoylation	
	EhNCABP166	EHI_093850	51 - 55	LKDLQDG IILSR YLQIETG	60.206	SUMO interaction	
			111 - 115	MNGEPKP IITLM FYLMYRF	61.066	SUMO interaction	
			129 - 133	FRLNDTP VRIID FSKWIKC	73.317	SUMO interaction	
			152	NNPLFDLKTEFSDGI	28.859	Sumoylation	[7]
			340	LEAMELVKEESSTPI	45.139	Sumoylation	

	455	ELIIQQLKDEIIQLT	25.181	Sumoylation
	503 - 507	IQKKEQE IIDIK KKNEETI	79.148	SUMO interaction
	514 - 518	KKKNEET IQLIQ KEMEKER	64.693	SUMO interaction
	585	QQELNKIKEENNEYK	54.425	Sumoylation
	645	EIKEKEVKELQKIIE	38.869	Sumoylation
	721	MKELERIKGEIEVLK	41.473	Sumoylation
	724 - 728	LERIKGE IEVLK IKETGDS	61.956	SUMO interaction
	749 - 753	IEELKKQ IEIIK NDNEKER	61.44	SUMO interaction
	877 - 881	INELNKS IIELK EEWNKKE	59.355	SUMO interaction
	881	NKSIIELKEEWNKKE	26.249	Sumoylation
	923	NENLKKVKEEIEKKT	43.876	Sumoylation
	1016	ENENEIIKKENKKKE	49.683	Sumoylation
	1058 - 1062	ERELGTK IKLIE MIKNEKD	60.142	SUMO interaction
	1065	IKLIEMIKNEKDIME	45.305	Sumoylation
	1076	DIMEKDFKKEVDNKN	18.489	Sumoylation
	1100 - 1104	IEKKKND ITLII QKNDEDK	66.441	SUMO interaction
	1129	NQEIEKIKSEKNDVQ	47.32	Sumoylation
	1168	ENEKLQIKEEHENNI	43.513	Sumoylation
	1228	TKKYEEVKGQIDGAH	40.95	Sumoylation
	1242	HQEVENIKKETEQQI	42.417	Sumoylation
	1253	EQQINQMKKECDEMQ	29.943	Sumoylation
	1267	QKTTFEAKEELRVQV	16.67	Sumoylation

			1309	ERTIEKLKNERNEKE	26.905	Sumoylation	
			1354	VMPKEVLKIDNTMLY	37.908	Sumoylation	
	Myosin heavy chains 1	EHI_140720	7-11	*MNKEEA ISIIQ SAIKEYC	67.784	SUMO interaction	[8]
			69	QNENAKLKNELQTKE	26.214	Sumoylation	
			183	ELADVKIKLDTTQQE	36.796	Sumoylation	
			366 - 370	KQKGDEE IIKLH DDLAEQA	62.69	SUMO interaction	
			463	AMDKELMKEEKLKQE	30.951	Sumoylation	
			468	LMKEEKLKQEIQALK	26.856	Sumoylation	
			711	DKEETIAKLEKDKNT	16.977	Sumoylation	
			787	RLDIVDLKGELESKE	25.353	Sumoylation	
			934 - 938	VADLENQ LEIIK DSIEEKD	62.341	SUMO interaction	
			994	KSKYDQLKSDNLMVL	38.597	Sumoylation	
			998 - 1002	DQLKSDN LMVLS EKEDIEE	61.696	SUMO interaction	
			1015	EEELSSVKEEMTKME	44.303	Sumoylation	
			1134	DDDYVRMKADNDKIR	38.706	Sumoylation	
Cholesterol trafficking	Phagosome-associated TMK96 (PATMK)	EHI_167650	17 - 21	YILLFLS IEIVG EYCDWNK	67.129	SUMO interaction	
			49 - 53	FNYNKTG VFIIR NRETETM	61.818	SUMO interaction	
			100 - 104	KELNTSF IVLIN DTTINGD	62.47	SUMO interaction	
			222	PSVLTILKQEVVNST	27.292	SUMOylation	
			483	NQTRQCVKTDIHQEI	37.543	SUMOylation	
			818 - 822	SQCSQCS IGYIV NYNGTRC	60.948	SUMO interaction	[9]

			951 - 955	YELTVSP ITIIL KKNEGCI	75.893	SUMO interaction	
			1082 - 1086	AVINKNN VSIIT EYAPYGS	61.53	SUMO interaction	
			1143 - 1147	RDIKLAN VLIIS LEDEMEI	67.864	SUMO interaction	
			1254 - 1258	ISLKIKS LLDLM WCQNPEN	61.691	SUMO interaction	
	EhNPC1	EHI_080220	325 - 329	IGFIIMG IIVVG FPMEGVI	64.822	SUMO interaction	[10]
			391 - 395	DIIDSAK VSILG QEESCEE	66.998	SUMO interaction	
			431 - 435	FLWKFKW IILTV VILCCIV	63.514	SUMO interaction	
			504 - 508	NQVLTQP LIVQL QQMIDEI	60.057	SUMO interaction	
			758 - 762	LAIGVDN IFILT NTIDEQP	66.525	SUMO interaction	
			1133	MNSIFDVKTFPYAYH	40.095	Sumoylation	
			1150 - 1154	YFQQYFN IVDLC VMDVCLA	60.804	SUMO interaction	
			1191 - 1195	LCVLMCV IDLIG IMYLWGV	60.091	SUMO interaction	
	EhNPC2a	EHI_068260	104	LPGPISIKFEMEIPN	42.327	Sumoylation	[10]
	EhNPC2b	EHI_188770	143 - 146	SCSEFGP VLVK* *******	59.366	SUMO interaction	[10]
			146	EFGPVLVK*******	37.397	Sumoylation	
Kinases	Phosphatidylinositol phosphate kinase	EHI_153770	49	GKTLPELKNEKYIFK	25.986	Sumoylation	[11]
			151	FVIKVIPKREEKILC	21.671	Sumoylation	
	p21-activated kinase 8 protein kinase	EHI_103610	103 - 107	TLCEQPS ILLLM QNESDFN	62.875	SUMO interaction	[12]

			180 - 184	AVNGEIP LTILP FSDNVEI	65.13	SUMO interaction	
			312	QAHIEKPKKEPPPEE	21.484	Sumoylation	
	EhC2PK	EHI_094060	67	KPKMRVAKLENTEQA	16.205	Sumoylation	[13]
			262 - 266	PGLKTGE VLVPQ EFTVTAK	59.917	SUMO interaction	
Calcium related proteins	EhCaBP1	EHI_120900	33	VSKKRAIKNEQLLQL	46.539	Sumoylation	[14]
	Cation/calcium exchanger	EHI_001770	46 - 50	VSFLINY LELLY CYNSYWI	59.298	SUMO interaction	[15]
			57 - 61	YCYNSYW IVLVC MVIGIII	62.999	SUMO interaction	
			196 - 200	HSIIFIL IYILY VCFIGYI	60.578	SUMO interaction	
			336 - 340	PFILSIG IILII HFTETQI	66.662	SUMO interaction	
Stress protein	Heat shock protein 70	EHI_002560	27	VAYFDVVKGEPVILQ	45.554	Sumoylation	[16]
			230	IINDNYIKVESSGGD	48.879	Sumoylation	
			287	NKKLIRLKKEAERIK	26.186	Sumoylation	
			294	KKEAERIKIELSGKP	42.012	Sumoylation	
			300	IKIELSGKPDAELDL	36.684	Sumoylation	
			366 - 370	KKGNVQL VLLIG GTCEMPR	61.124	SUMO interaction	
			423 - 427	NSICSKF IYDIV PTPIGIE	64.333	SUMO interaction	
Adhesin	Gal/GalNAc lectin heavy subunit	EHI_012270	206 - 210	TNTTSKC IIDVH KNNVNTH	60.701	SUMO interaction	[17]
			218 - 222	KNNVNTH LAIIL GITDSTV	60.45	SUMO interaction	
			255	GVTIYYLKGDSYATD	37.476	Sumoylation	
			270	NIKLKDLKYETLVKY	26.485	Sumoylation	

	437	PKAKPKAKKECPATC	16.996	Sumoylation
	552	VQEKVCVKTSPYIEM	41.585	Sumoylation
	970 - 974	IECKIQE IVITE KDGIKTT	60.791	SUMO interaction

The access number and sequence of proteins (already identified) that participate in *Entamoeba histolytica* phagocytosis were obtained from KEGG (https://www.genome.jp/kegg, 29/04/2021), AmoebaDB (https://amoebadb.org/amoeba/app, 29/04/2021) and NCBI (https://www.ncbi.nlm.nih.gov, 29/04/2021) databases. Then, these proteins were analysed using the GPS-SUMO 1.0 (http://sumosp.biocuckoo.org, 29/04/2021) program to predict SUMOylation sites.

The results displayed several hypothetical sites in the studied proteins, suggesting that this posttranslational modification could regulate their function during this virulence event.

[1] Y. S.-N. Kumiko Nakada-Tsukui and V. A. and T. Nozaki, "A Retromerlike Complex Is a Novel Rab7 Effector That Is Involved in the Transport of the Virulence Factor Cysteine Protease in the Enteric Protozoan Parasite Entamoeba histolytica," *Mol. Biol. Cell*, vol. 16, no. November, pp. 5356–5372, 2005, doi: 10.1091/mbc.E05.

[2] I. Lpez-Reyes *et al.*, "Detection of the endosomal sorting complex required for transport in entamoeba histolytica and characterization of the EhVps4 protein," *J. Biomed. Biotechnol.*, vol. 2010, pp. 1–15, 2010, doi: 10.1155/2010/890674.

[3] R. Javier-Reyna, S. Montaño, G. García-Rivera, M. A. Rodríguez, A. González-Robles, and E. Orozco, "EhRabB mobilises the EhCPADH complex through the actin cytoskeleton during phagocytosis of Entamoeba histolytica," *Cell. Microbiol.*, vol. 21, no. 10, 2019, doi: 10.1111/cmi.13071.

[4] Y. Saito-Nakano, R. Wahyuni, K. Nakada-Tsukui, K. Tomii, and T. Nozaki, "Rab7D small GTPase is involved in phago-, trogocytosis and cytoskeletal reorganization in the enteric protozoan Entamoeba histolytica," *Cell. Microbiol.*, vol. 23, no. 1, 2021, doi: 10.1111/cmi.13267.

[5] M. Babuta, M. S. Mansuri, S. Bhattacharya, and A. Bhattacharya, "The Entamoeba histolytica, Arp2/3 Complex Is Recruited to Phagocytic Cups through an Atypical Kinase EhAK1," *PLoS Pathog.*, vol. 11, no. 12, pp. 1–27, 2015, doi: 10.1371/journal.ppat.1005310.

[6] A. Sateriale, A. Vaithilingam, L. Donnelly, P. Miller, and C. D. Huston, "Feed-forward regulation of phagocytosis by entamoeba histolytica," *Infect. Immun.*, vol. 80, no. 12, pp. 4456–4462, 2012, doi: 10.1128/IAI.00671-12.

[7] A. D. Campos-Parra, N. A. Hernández-Cuevas, R. Hernandez-Rivas, and M. Vargas, "EhNCABP166: A nucleocytoplasmic actin-binding protein from Entamoeba histolytica," *Mol. Biochem. Parasitol.*, vol. 172, no. 1, pp. 19–30, 2010, doi: 10.1016/j.molbiopara.2010.03.010.

[8] H. Voigt, J. C. Olivo, P. Sansonetti, and N. Guillén, "Myosin IB from Entamoeba histolytica is involved in phagocytosis of human erythrocytes," *J. Cell Sci.*, vol. 112, no. 8, pp. 1191–1201, 1999.

[9] D. R. Boettner *et al.*, "Entamoeba histolytica phagocytosis of human erythrocytes involves PATMK, a member of the transmembrane kinase family," *PLoS Pathog.*, vol. 4, no. 1, pp. 0122–0133, 2008, doi: 10.1371/journal.ppat.0040008.

[10] J. Bolaños *et al.*, "EhNPC1 and EhNPC2 Proteins Participate in Trafficking of Exogenous Cholesterol in Entamoeba histolytica Trophozoites: Relevance for Phagocytosis," *PLoS Pathog.*, vol. 12, no. 12, pp. 1–29, 2016, doi: 10.1371/journal.ppat.1006089.

[11] S. Sharma, S. Bhattacharya, and A. Bhattacharya, "PtdIns(4,5)P2 is generated by a novel phosphatidylinositol 4-phosphate 5-kinase in the protist parasite Entamoeba histolytica," *FEBS J.*, vol. 286, no. 11, pp. 2216–2234, 2019, doi: 10.1111/febs.14804.

[12] E. Labruyère, C. Zimmer, V. Galy, J. C. Olivo-Marin, and N. Guillén, "EhPAK, a member of the p21-activated kinase family, is involved in the control of Entamoeba histolytica migration and phagocytosis," *J. Cell Sci.*, vol. 116, no. 1, pp. 61–71, 2003, doi: 10.1242/jcs.00190.

[13] Somlata, S. Bhattacharya, and A. Bhattacharya, "A C2 domain protein kinase initiates phagocytosis in the protozoan parasite Entamoeba histolytica," *Nat. Commun.*, vol. 2, no. 1, pp. 210–230, 2011, doi: 10.1038/ncomms1199.

[14] S. Kumar *et al.*, "Crystal structure and trimer-monomer transition of N-terminal domain of EhCaBP1 from entamoeba histolytica," *Biophys. J.*, vol. 98, no. 12, pp. 2933–2942, 2010, doi: 10.1016/j.bpj.2010.03.048.

[15] M. Valle-Solis *et al.*, "A Calcium/Cation Exchanger Participates in the Programmed Cell Death and in vitro Virulence of Entamoeba histolytica," *Front. Cell. Infect. Microbiol.*, vol. 8, no. October, p. 342, 2018, doi: 10.3389/fcimb.2018.00342.

[16] O. S. b and M. D. Stephan Ortner, Barbara Plaimauer, Marina Binder, Gerhard Wiedermann, "Humoral immune response against a 70-kilodalton heat shock protein of Entamoeba histolytica in a group of patients with invasive amoebiasis," *Trends Parasitol.*, vol. 54, pp. 175–183, 2021, doi: 10.1016/j.pt.2021.01.001.

[17] B. J. Mann, "Structure and function of the Entamoeba histolytica Gal/GalNAc lectin," *Int. Rev. Cytol.*, vol. 216, pp. 59–80, 2002, doi: 10.1016/S0074-7696(02)16003-7.
